# Recent Meta-Analyses in the Clinical High Risk for Psychosis Population: Clinical Interpretation of Findings and Suggestions for Future Research

**DOI:** 10.3389/fpsyt.2018.00502

**Published:** 2018-10-12

**Authors:** Barnaby Nelson, Günter Paul Amminger, Patrick Denistoon McGorry

**Affiliations:** ^1^Orygen, The National Centre of Excellence in Youth Mental Health, University of Melbourne, Parkville, VIC, Australia; ^2^Centre for Youth Mental Health, The University of Melbourne, Melbourne, VIC, Australia

**Keywords:** psychosis, prevention, ultra high risk for psychosis, schizophrenia, meta - analysis

One of the major advances over the past 20 years in psychiatry is the capacity not only to identify people at incipient risk for psychosis and other disabling mental disorders, but also to improve their levels of distress and functioning and reduce their risk of progression to sustained psychotic disorder, at least while treatment is being provided and for at least 1–2 years from baseline. These statements are supported by level 1 Cochrane evidence and one would expect this progress to be understood and valued by a field in need of positive findings pointing to better outcomes. It is therefore puzzling why recent meta-analyses appear to have focused on second order issues and in doing so have distracted from the key message of this research literature and fuelled the traditional skepticism and pessimism with which our discipline is so replete.

The latest example of this phenomenon are the two network meta-analyses by Davies et al. (1, 2) of preventive interventions in the clinical high risk (CHR, otherwise termed ultra-high risk) for psychosis population. The primary focus of the first meta-analysis (1) is transition to psychosis rates in these trials, with a secondary focus on acceptability of treatments, operationalised as study drop-out due to any cause. The analysis showed no significant efficacy of any one particular intervention over others at 6 and 12 months on either outcome. The authors conclude from their analysis that there is no evidence that any specific intervention is more effective than any other trialed intervention in preventing onset of psychosis and that “individuals meeting CHR-P criteria may be informed that, at present, there is no evidence for specific treatments being more effective than any others, and current options should be carefully weighted on a personal basis depending on an individual's needs” (p.206). The second meta-analysis (2) focused on attenuated positive psychotic symptoms as an outcome, with similar conclusions.

As a general point, this message of how to present current evidence to patients in the clinical context seems to be negatively weighted and omits the fact that the trials indicate in group-level analysis that most CHR patients *improve* in their symptoms and functioning over time and transition rates are reduced. While it may well be true that the field has not yet identified a single specific intervention that is more effective than others (a substantial challenge given the clinical heterogeneity of the CHR population, as the authors note), the trials do show that most patients improve in response to treatment provided in specialist research clinics and transitions are at least delayed. There is of course an important sub-group who manifest persistent symptoms and functional difficulties that do not respond to treatment. The question of whether targeted trial interventions *pooled together* yield improved outcomes compared to control groups, as suggested by previous meta-analyses (3–5), is in fact not directly addressed in these current meta-analyses. While different cognitive behavior therapy (CBT) protocols were pooled together and compared to needs-based intervention (NBI) and different antipsychotic treatments were pooled together and compared to NBI in the first meta-analysis (and only pooled antipsychotic treatments in the second meta-analysis), the authors do not seem to have pooled together all trial interventions (both psychosocial and pharmacological) in comparison with NBI. If this had been done, similar findings to previous meta analyses (3–5) may well have emerged.

Another important methodological observation needs to be made. A curious aspect of both meta-analyses is that McGorry et al “Neurapro” trial (6) has been categorized as “omega-3 and NBI or placebo and NBI.” However, the psychosocial intervention provided in this trial was combined CBT and case management, termed cognitive-behavioral case management (CBCM), i.e., omega-3+NBI+CBT vs. placebo+NBI+CBT (6– 8). The nature of this psychosocial intervention was in fact critical to the interpretation of the trial's negative outcome, discussed in detail elsewhere (6, 9, 10). In brief, the manualised CBCM received by both treatment groups (as well as the use of antidepressant medications in both groups) may have been sufficiently effective to have produced a ceiling effect beyond which there was no scope for omega-3 polyunsaturated fatty acids (PUFA) to confer additional benefit. This may have interfered with being able to properly test the efficacy of omega-3 PUFA. This possibility is consistent with the fact that the placebo group in the original omega-3 PUFA trial, also included in the current meta-analyses, failed to show the level of symptomatic and functional improvement seen in the Neurapro trial. The classification of the intervention provided in that trial as NBI rather than as CBCM, while presumably guided by the aim of increasing the statistical power for the omega-3 comparison, may have had a substantial impact on the meta-analytical findings, given that it is the largest trial included in the meta-analyses (*n* = 304) and may have inflated the effect of NBI (making it statistically more difficult to find benefit in favor of any of the specific interventions). Indeed, when this trial was removed from the second meta-analysis in order to conduct sensitivity analyses, CBT-F plus NBI emerged as significantly more effective than NBI alone at 12 months on the primary outcome (reduction of attenuated positive psychotic symptoms). This is consistent with our speculation that if this trial had been coded as CBT rather than as NBI, NBI may have had a weaker effect in the analyses and other interventions, most likely CBT, may well have demonstrated a superior effect.

It also strikes us that it would have been important to include functioning and the range of clinical outcomes (depression, general psychopathology, etc.) as an outcome in these meta-analyses, particularly as these are often key targets of the psychosocial interventions and secondary outcomes of the trials included. Not including these outcomes seems to ignore critical information and leaves us with the unanswered question of whether any of the specific interventions had a positive effect on these other clinical outcomes, even if the interventions were not associated with reduced transition rate, attenuated psychotic symptoms or increased acceptability of treatment. Clearly, a positive effect of any of the interventions on these outcomes would have important clinical implications. We note that Devoe et al.'s (11, 12) meta analysis of the effect of trial interventions on negative symptoms in UHR studies found a trend-level positive benefit for *N*-methyl-D-aspartate-receptor (NMDAR) modulators compared to placebo.

We agree with Davies et al that enrichment strategies need to be pursued to guard against under-powered trials. However, another strategy is to take the approach of developing interventions that respond to the evolving clinical profile or treatment response of patients (“adaptive interventions”), such as sequential multiple assignment randomized trials (SMART trials) (13). These are interventions in which the type or dosage of treatment is individualized on the basis of patient characteristics, such as psychological features, clinical presentation or mechanism-linked biomarkers, and then is repeatedly adjusted over time in response to patient progress. Interventions can also be tailored at critical decision points according to response or other patient characteristics, such as specific biomarker changes or comorbidity, and also patient preference. This approach has the advantage of providing more intensive treatment for those with persistent symptoms or functional difficulties, rather than simply continuing with the same treatment regimen, thereby mimicing what tends to occur in standard clinical practice (and may therefore yield findings that are more useful for clinical translation). It also has the effect of enriching the sample for psychosis risk because those who do not respond to initial treatment steps (i.e., those who are not “rapid responders”) are likely to constitute a sub-group at increased risk for fully-fledged psychosis in whom further specific treatments can be trialed.

Finally, it may be of value to not only test interventions that target specific sub-groups within the UHR population based on putative mechanisms in that sub-group (a form of precision medicine), but also to conduct trials in young people at transdiagnostic risk. In other words, the aspiration to “narrow” the treatment target can be complemented by a broadening of clinical population and intervention strategies; as we have recently argued (14), these strategies are not mutually exclusive. Trialing interventions in a broad at-risk group is consistent with the diffuse, overlapping clinical presentations seen in early stages of disorder [the “problem of comorbidity” (15–17)]. The identification of biopsychosocial mechanisms driving the onset of disorder and developing effective interventions in this group need to evolve in parallel and inform each other. While progress with identifying causal mechanisms can certainly guide treatment targets, waiting for these mechanisms to be identified before testing preventive treatments does patients a disservice, particularly if the cost-benefit balance of these treatments is favorable. In turn, effective treatments can, in the spirit of reverse engineering, shed light on the pathogenesis of disorder.

## Author contributions

All authors listed have made a substantial, direct and intellectual contribution to the work, and approved it for publication.

### Conflict of interest statement

The authors declare that the research was conducted in the absence of any commercial or financial relationships that could be construed as a potential conflict of interest.
